# Bioinformatics Analysis Identifies Molecular Markers Regulating Development and Progression of Endometriosis and Potential Therapeutic Drugs

**DOI:** 10.3389/fgene.2021.622683

**Published:** 2021-08-04

**Authors:** Ying Peng, Cheng Peng, Zheng Fang, Gang Chen

**Affiliations:** Department of Obstetrics and Gynecology, The First Affiliated Hospital of USTC, Division of Life Sciences and Medicine, University of Science and Technology of China, Hefei, China

**Keywords:** endometriosis, bioinformatics analysis, differentially expressed genes, immune mechanism, molecular markers, potential drugs

## Abstract

Endometriosis, a common disease that presents as polymorphism, invasiveness, and extensiveness, with clinical manifestations including dysmenorrhea, infertility, and menstrual abnormalities, seriously affects quality of life in women. To date, its underlying etiological mechanism of action and the associated regulatory genes remain unclear. This study aimed to identify molecular markers and elucidate mechanisms underlying the development and progression of endometriosis. Specifically, we downloaded five microarray expression datasets, namely, GSE11691, GSE23339, GSE25628, GSE7305, and GSE105764, from the Gene Expression Omnibus (GEO) database. These datasets, obtained from endometriosis tissues, alongside normal controls, were subjected to in-depth bioinformatics analysis for identification of differentially expressed genes (DEGs), followed by analysis of their function and pathways *via* gene ontology (GO) and KEGG pathway enrichment analyses. Moreover, we constructed a protein–protein interaction (PPI) network to explore the hub genes and modules, and then applied machine learning algorithms support vector machine-recursive feature elimination and least absolute shrinkage and selection operator (LASSO) analysis to identify key genes. Furthermore, we adopted the CIBERSORTx algorithm to estimate levels of immune cell infiltration while the connective map (CMAP) database was used to identify potential therapeutic drugs in endometriosis. As a result, a total of 423 DEGs, namely, 233 and 190 upregulated and downregulated, were identified. On the other hand, a total of 1,733 PPIs were obtained from the PPI network. The DEGs were mainly enriched in immune-related mechanisms. Furthermore, machine learning and LASSO algorithms identified three key genes, namely, apelin receptor (*APLNR*), C–C motif chemokine ligand 21 (*CCL21*), and Fc fragment of IgG receptor IIa (*FCGR2A*). Furthermore, 16 small molecular compounds associated with endometriosis treatment were identified, and their mechanism of action was also revealed. Taken together, the findings of this study provide new insights into the molecular factors regulating occurrence and progression of endometriosis and its underlying mechanism of action. The identified therapeutic drugs and molecular markers may have clinical significance in early diagnosis of endometriosis.

## Introduction

Endometriosis (EM), in which active endometrium is implanted in any site outside the uterine cavity, is a common gynecological disease characterized by chronic pelvic pain, dysmenorrhea, and infertility (Aghajanova and Giudice, 2010). Approximately 1% of all EM cases exhibit malignant transformation (Guo et al., 2012), which greatly reduces the quality of life of women at a childbearing age. Treatment of EM is challenging due to a high postoperative recurrence rate and the inhibitory effect of hormone drugs on ovarian function (Wu et al., 2017). Currently, surgical resection and hormone suppression represent the gold standard treatment for EM, although these are limited by high occurrence of side effects (Vercellini et al., 2013), recurrence rates, and treatment costs (Simoens et al., 2012). Therefore, identification of novel molecular factors, coupled with unraveling of the underlying mechanisms of EM will aid in development of effective treatment therapies.

Numerous hypotheses have been proposed to describe the underlying molecular mechanisms of EM, including the menstrual reflux theory (Kruitwagen et al., 1991), ectopic implantation, epigenetic as well as immune and inflammatory factors, immunodeficiency (Ahn et al., 2015), eutopic endometrium determinism, and stem cell factors (Burney and Giudice, 2012). To date, however, no single theory can explain all the causes of EM, although reflux and ectopic EM implantation represent the most commonly accepted theories. Notably, only a handful of women have EM (Hill et al., 2020). Advancement in molecular biology and pathophysiology technologies has revealed that pathogenesis of EM is closely associated with the body’s immune imbalance (Burney and Giudice, 2012). Previous studies have shown that cell-mediated and humoral immune changes contribute to disease progression in women with EM. Notably, functional changes in immune components, such as monocytes/macrophages, natural killer (NK) cells, T lymphocytes, B cells, and cytokines, in peritoneal fluid of women with EM have been described (Zou et al., 2021). Although numerous and brisk EMaICI, comprising several types of immune cells in all EM forms, have revealed acute immunological reactions within the microenvironment of EM lesions (Herington et al., 2011), the role of these changes in disease development remains unclear. Previous studies have demonstrated that overexpression and downregulation of antiapoptotic and proapoptotic factors, respectively, may interfere with peritoneal homeostasis (Erekat et al., 2018), while the mitogen-activated protein kinase (MAPK) signaling pathway has been shown to play a key role in disease occurrence (Lotfaliansaremi et al., 2020). Moreover, apoptosis pathways of Fas-FasL and tumor necrosis factor (TNF)-a seem to play a key role in immune monitoring of the peritoneal microenvironment (Yamada et al., 2017). Overall, these changes may prevent clearance of endometrial cells that reach the peritoneal cavity, thereby allowing their implantation and development. The current consensus is that changes in immune-related cell function play a crucial role in occurrence and progression of EM (Ahn et al., 2015).

Although numerous studies have reported occurrence of immune abnormalities, the role of the immune system in EM is not fully understood, although some evidence has demonstrated that impaired immune homeostasis is associated with increased implantation, proliferation, and angiogenesis of ectopic endometrium (Holtan et al., 2009). Although EM is a benign disease, EM cells exhibit many characteristics similar to those observed in malignant tumors, including migration and aggressiveness. Therefore, exploring immune dysfunction in EM may help elucidate its role in pathogenesis of the disease and generate relevant insights to guide development of new treatment strategies. In addition, with the rapid development of high-throughput sequencing technologies, more and more potential biomarkers associated with EM were identified. Liu et al. (2015) found that the EGF, IL-β and AGTR1 play an important role in the pathogenesis of EM by using the microarray dataset. Cui et al. (2020) employed three microarray datasets, constructed a network, and revealed that YAP/TAZ was upregulated, and MOB1, pMOB1, SAV1, LATS1, and LATS2 were downregulated in endometrium. Dai et al. (2019) using four microarray datasets (GSE11691, GSE23339, GSE25628, and GSE78851) identified a number of differentially expressed genes (DEGs). However, these studies lack in-depth analysis and have no external validation dataset to evaluate their expression level. Therefore, a comprehensive analysis of the potential biomarkers based on a number of microarray datasets is in urgent demand.

In the present study, we downloaded five microarray datasets (GSE11691, GSE23339, GSE25628, GSE105764, and GSE7305) from the Gene Expression Omnibus (GEO) database to explore the potential biomarkers. We employed in-depth bioinformatics analysis to identify candidate genes and key signaling pathways regulating occurrence and progression of EM, as well as possible therapeutic drugs for its treatment. Overall, these findings provide new insights into the underlying molecular mechanisms and potential candidate drugs for treatment of EM.

## Materials and Methods

### Data Collection

We employed the ‘‘GEOquery’’ package, implemented in R software version 3.6.1^1^ to download GSE11691, GSE23339, GSE25628, GSE105764, and GSE7305 datasets from the GEO database^2^ using “endometriosis” as the keyword (Davis and Meltzer, 2007). A total of 87 samples were obtained: GSE11691 contained 9 healthy and 9 diseased samples (Hull et al., 2008), GSE105764 included 8 EM samples and 8 normal samples (Zhao et al., 2018), GSE23339 comprised 9 and 10 healthy and diseased samples (Hawkins et al., 2011), GSE7305 consisted of 10 diseased samples and 10 healthy samples (Hever et al., 2007), while GSE25628 contained 6 healthy and 8 diseased samples (Heydari et al., 2020). Among them, GSE11691, GSE23339, and GSE25628 served as a training dataset, while GSE7305 and GSE105764 were used as an external dataset.

### Data Preprocessing and Differential Gene Expression Analysis

The batch effect in the training dataset was removed using the ‘‘sva’’ R package.^3^ The effect of the correction between samples was calculated by two-dimensional principal component analysis (PCA) clustering. DEGs were screened using “limma” R package and the volcano plots of the DEGs were generated using “ggplot2” to show differential expression patterns (Ritchie et al., 2015). Additionally, the genes with adjusted *p*-value < 0.05 and | log2FC| > 1 were considered statistically significant.

### Gene Set Enrichment Analysis

Functional and KEGG pathway enrichment analyses of the DEGs were performed using “clusterProfiler” R package (Yu, 2018). The significant pathways were identified with the adjusted *p*-value < 0.05.

### Construction of a Protein–Protein Interaction Network

To construct a protein--protein interaction (PPI) network, we uploaded all the DEGs to the STRING protein database.^4^ The gene interaction score > 0.7 was used to constructed a network. We then used the MCODE algorithm and cytoscape software to identify and visualize the hub networks and hub genes (Bader and Hogue, 2003; Shannon et al., 2003). As a result, the top two hub networks that contained 22 hub genes were subsequently used for the downstream analysis.

### Screening and Verification of Key Genes

The key genes were identified using least absolute shrinkage and selection operator (LASSO) regression analysis and support vector machine (SVM) analysis based on the genes of the top two subnetworks, respectively. The LASSO is a dimension-reduction algorithm and showed a higher superiority compared to regression analysis (Tibshirani, 1996). The support vector machine-recursive feature elimination (SVM-RFE) was a machine learning algorithm that was applied to identify the best variables *via* eliminating feature vectors generated by SVM and further demonstrated the biomarkers in EM through the “e1071” R package (Sanz et al., 2018). To further evaluate the expression profile, we used the GSE7305 and GSE105764 dataset as an external dataset. The *p*-value was calculated using the Wilcoxon rank test analysis. In addition, we also used the ‘‘pROC’’^5^ to estimate the performance between EM and normal tissue.

### Functional Identification of Key Genes

To further investigate functions of the key genes, we performed gene set enrichment analysis (GSEA) analysis based on each key gene’s expression profile and then classified them into high and low expression groups based on median gene expression levels. The significant pathways were identified based on the *p*-value < 0.05.

### Correlation Between Gene Expression and Immunity

We uploaded the expression data for all training dataset samples to the CIBERSORTx^6^ and calculated the 22 immune cells’ infiltration level (Newman et al., 2019). We then performed correlation analysis between the 22 immune cells and the key genes.

### Identification of Potential Drugs

To identify potential therapeutic drugs for EM treatment, we uploaded the DEGs to the connective map (CMAP) database^7^ and then applied enrichment analysis to pull out significantly enriched drugs (Musa et al., 2017). Generally, a negative enrichment value indicates that the drug is more likely to treat the disease, with a greater magnitude of the enrichment value implying more relevance of the drug to the disease. Therefore, we further screened for drugs with an enrichment of less than -0.5.

## Results

### DEGs in EM

**FIGURE 1:**
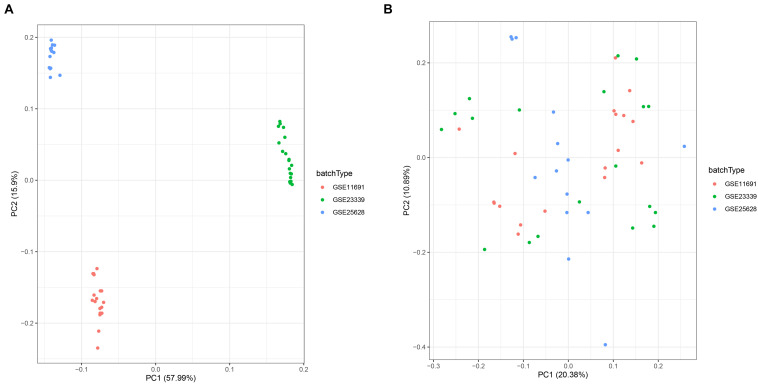
Principal component analysis (PCA) showing patterns of gene expression across datasets. Points of the scatter plots represent samples based on the top two principal components (PC1 and PC2) of gene expression profiles without **(A)** and with **(B)** the removal of batch effect. Colors denote corresponding samples across three different datasets.

**FIGURE 2:**
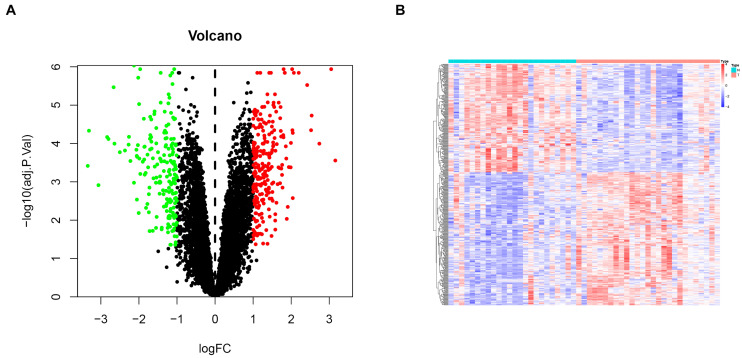
Differential gene expression analysis in endometriosis cases. **(A)** Volcanic plots for differentially expressed genes. Red and green dots denote significantly upregulated and downregulated genes, respectively, while black dots indicate non-significant genes. **(B)** A heat map showing profiles of the differentially expressed genes.

To comprehensively investigate gene expression in EM, we first integrated three datasets, namely GSE11691, GSE23339, and GSE25628, into a complete dataset after removing batch effects using the “sva” R package. We then used PCA analysis to determine the distribution of the samples before and after correction. Figure 1A shows that the distribution of the three datasets before batch effect was removed, and Figure 1B confirms that all confounding factors were successfully removed from the corrected samples (Figures 1A,B). In addition, The Differential expression analysis revealed a total of 423 DEGs across the combined dataset, namely, 233 and 190 upregulated and downregulated genes (Figures 2A,B).

### Function and Pathway Enrichment of the DEGs

**FIGURE 3:**
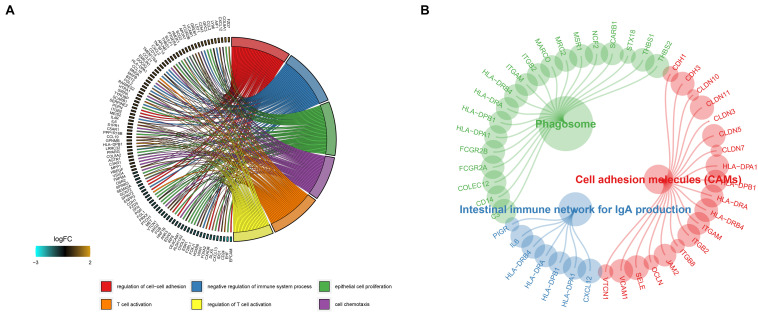
Gene ontology and KEGG enrichment analysis results of the DEGs are shown in panels **(A,B)**.

To investigate the molecular function and pathways that are involved in the DEGs, we then conducted an enrichment analysis using the clusterProfiler R package. Based on the screening criterion, we discovered that the genes are mainly involved in T cell activation, regulation T cell activation, and cell chemotaxis in gene ontology (GO) results (Figure 3A), while phagosome, cell adhesion molecules (CAMs), and the intestinal immune network for IgA production pathway were enriched in KEGG results (Figure 3B).

### Construction of PPI Networks

**FIGURE 4:**
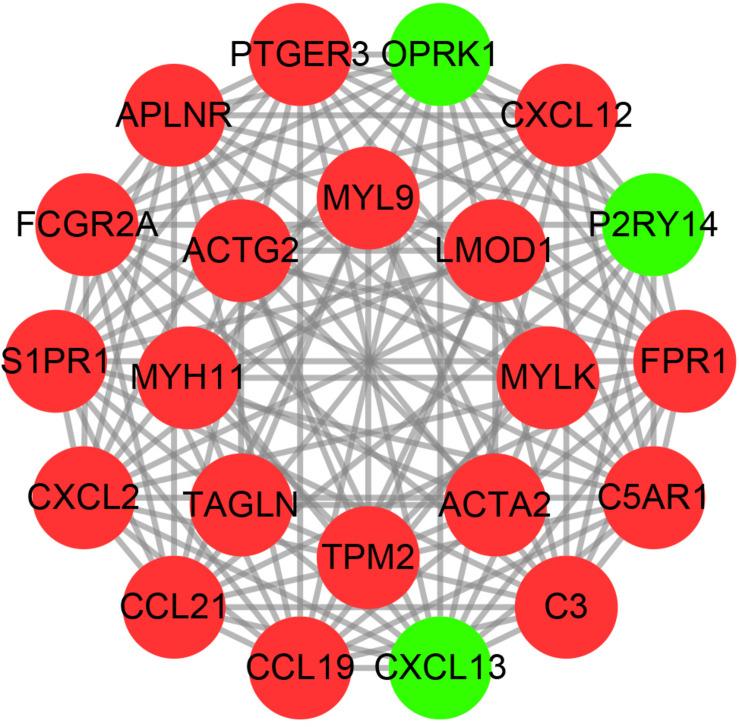
The hub PPI networks of the DEGs. Each circle represents the genes, while the lines denote the interaction among proteins from different genes. Red and green dots indicate relatively upregulated and downregulated genes, respectively.

We used the string online database to explore the interaction network of all DEGs and further visualized it using the cytoscape software. We then applied the MCODE algorithm to explore the hub networks and genes. As a result, the top two subnetworks with 22 hub genes were identified, namely, CXCL13, CCL19, APLNR, PTGER3, P2RY14, OPRK1, CCL21, FPR1, CXCL2, FCGR2A, S1PR1, C3, CXCL12, C5AR1, TAGLN, LMOD1, TPM2, ACTG2, MYL9, MYH11, MYLK, and ACTA2 (Figure 4).

### Key Genes and Enriched Pathways in EM

**FIGURE 5:**
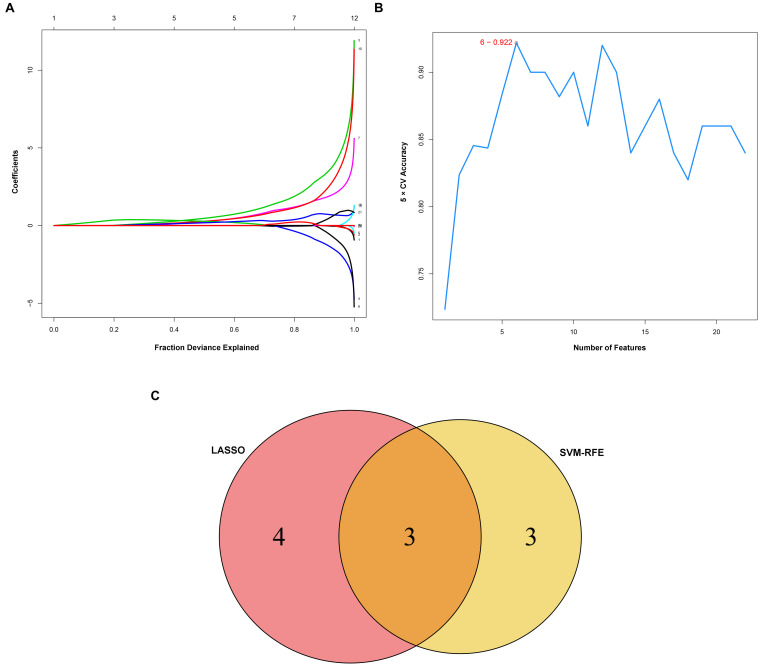
Identification and verification of diagnostic markers. **(A)** Diagnostic markers identified using the least absolute shrinkage and selection operator (LASSO) logistic regression algorithm. Different colors represent different genes. **(B)** Support vector machine-recursive feature elimination (SVM-RFE) algorithm to screen diagnostic markers. **(C)** Venn diagram showing the intersection among diagnostic markers between the two algorithms.

**FIGURE 6:**
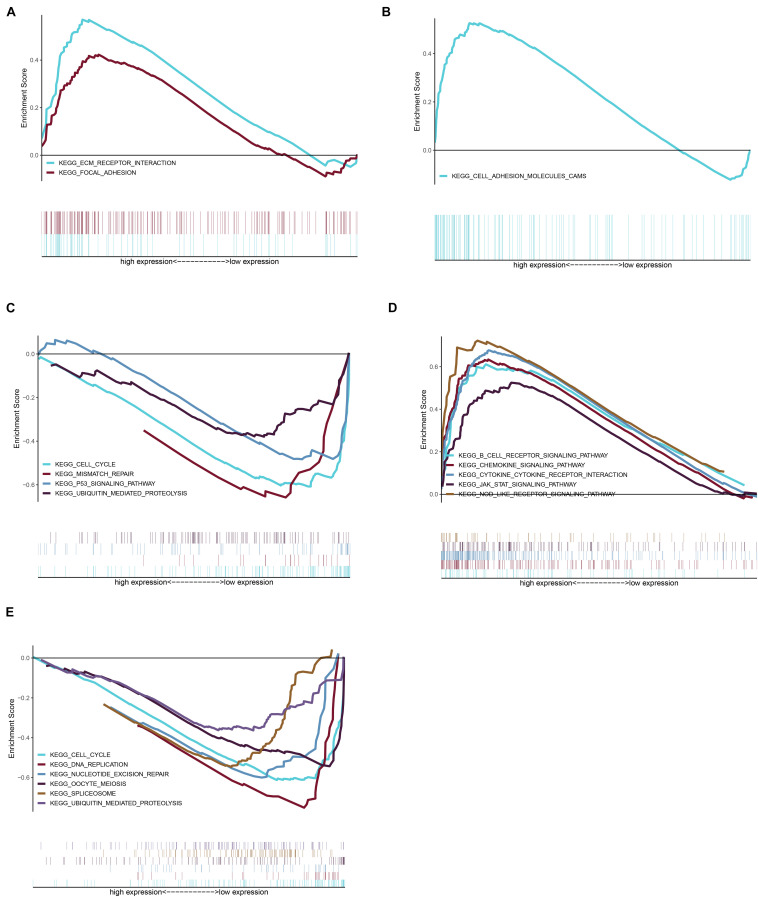
Gene set enrichment analysis (GSEA) was performed for the three key genes based on the median expression profile. **(A)** The significant pathways were enriched in the high expression group of APLNR. **(B,C)** The significant pathways were enriched in the high and low expression group of CCL21, respectively. **(D,E)** In the FCGR2A, the significant pathway is shown in the high expression and low expression group, respectively.

**FIGURE 7:**
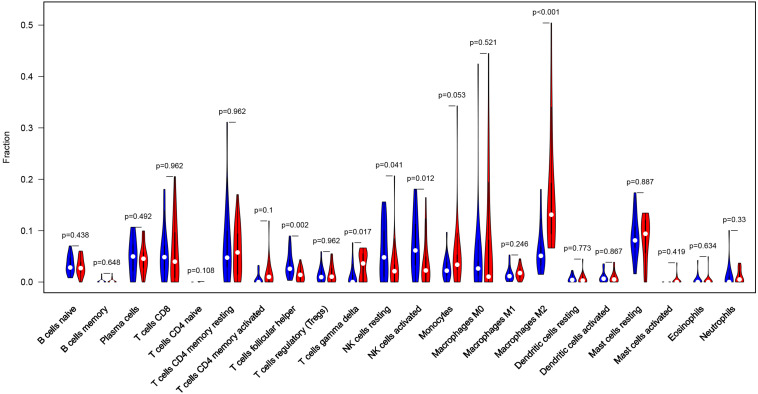
Profile of the level of 22 immune cells’ infiltration in the EM and normal tissues.

**FIGURE 8:**
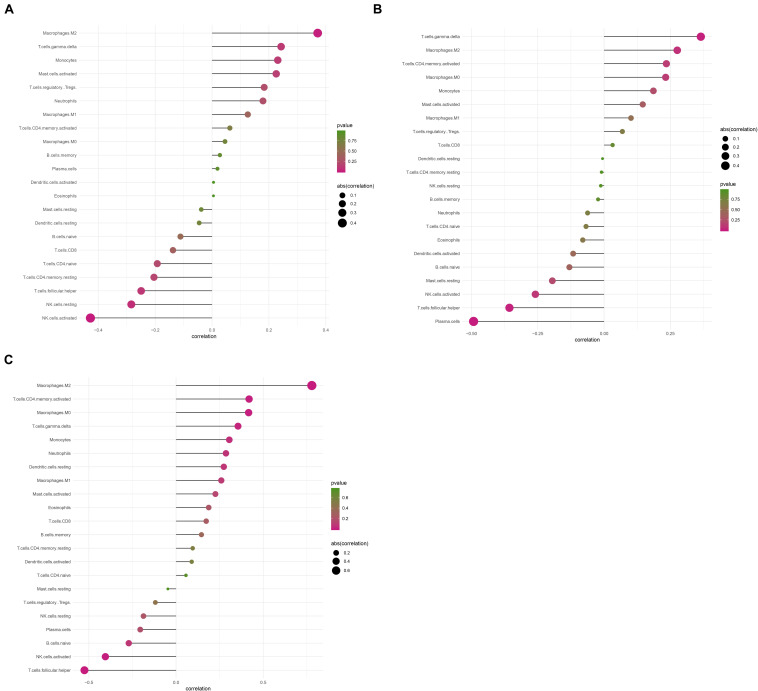
The correlation between levels of immune cell infiltration with expression of three key genes. **(A)** Correlation between APLNR and 22 immune cells. **(B)** Correlation between FCGR2A and 22 immune cells. **(C)** Correlation between CCL21 and 22 immune cells. The size of the dots represents the strength of the correlation between genes and immune cells; with larger dots implying a stronger correlation, and vice versa. The color of the dots represents the *p*-value.

Considering too much hub genes were identified from the hub networks, we then used the LASSO regression analysis and SVM-RFE analysis to make a feature selection. We firstly used the LASSO analysis to screen the key genes from the 22 hub genes, and 7 genes, namely, CXCL13, APLNR, CCL21, FCGR2A, CXCL12, TAGLN and ACTA2, were identified (Figure 5A). Three genes including APLNR, CCL21, and FCGR2A were identified from 22 hub genes by the SVM-RFE analysis (Figure 5B). The combined results from LASSO regression and SVM-RFE analyses revealed three key genes in ME, namely, apelin receptor (APLNR), C–C motif chemokine ligand 21 (CCL21), and Fc fragment of IgG receptor IIa (FCGR2A) (Figure 5C). GSEA analysis of expression of the three genes in APLNR revealed significant enrichment of focal adhesion and ECM–receptor interaction in the high expression group, and none in the low expression group (Figure 6A). In the CCL21 group, genes in the high expression group were significantly enriched in the CAMs pathway, whereas those in the low expression group were significantly enriched in cell cycle, mismatch repair, P53 signaling pathway, and ubiquitin-mediated proteolysis (Figures 6B,C). In the FCGR2A group, the high expression group was significantly enriched in B cell receptor and chemokine signaling pathways, cytokine–cytokine receptor interaction, and Jak Stat and NOD-like receptor signaling pathways. On the other hand, genes in the low expression group were significantly enriched in cell cycle, DNA replication, nucleotide excision repair, oocyte meiosis, spliceosome, and ubiquitin-mediated proteolysis pathways (Figures 6D,E). In addition, we also adopted the CIBERSORTx algorithm to explore levels of 22 immune cells’ infiltration, and found that T follicular helper cells, gamma delta T cells, resting NK cells, activated NK cells, and M2 macrophages have a significant difference between normal and EM tissues (Figure 7). Since these three genes were significantly involved in immune-related pathways, we further evaluated their association with immune cells. Results showed that APLNR was most positively and negatively correlated with M2 macrophages and activated NK cells, respectively. On the other hand, CCL21 was most positively and negatively correlated with gamma delta T cells and plasma cells, respectively. Similarly, FCGR2A was most positively and negatively correlated with M2 macrophages and follicular helper T cells, respectively (Figure 8).

### Validation of the Key Genes

**FIGURE 9:**
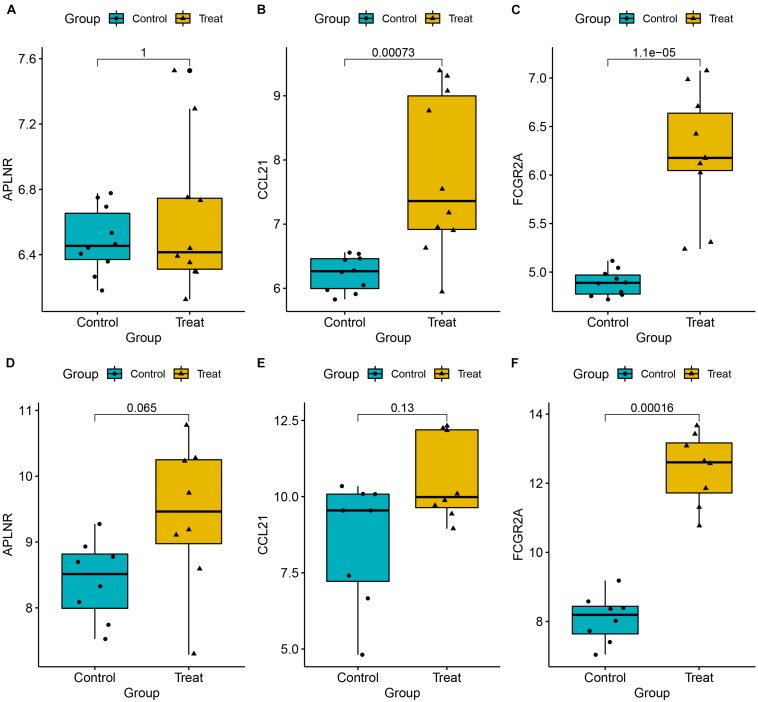
Expression levels for three key genes, namely APLNR **(A,D)**, CCL21 **(B,E)**, and CCL21 **(C,F)**, between EM and normal tissues. Validation was done using two external datasets (GSE7305 and GSE105764).

To ensure reliable and stability of the results, we further validated expression of the three key genes using two external datasets (GSE7305 and GSE105764). Results revealed that CCL21 and FCGR2A were significantly upregulated in diseased relative to normal tissues. Conversely, APLNR was slightly, but not significantly, upregulated in diseased relative to normal tissues. Overall, these results corroborated the above findings, thereby affirming their reliability (Figures 9A–F). Moreover, to assess the discrimination power of the three genes in the EM and normal tissue, we also performed a ROC analysis on the two external datasets. Interestingly, the three genes showed a high performance to distinguish the EM and normal tissue, indicating that the three genes have potential as a diagnostic biomarker (Supplementary Figure 1).

### Identification of Small Molecular Compounds for Treatment of EM

**FIGURE 10:**
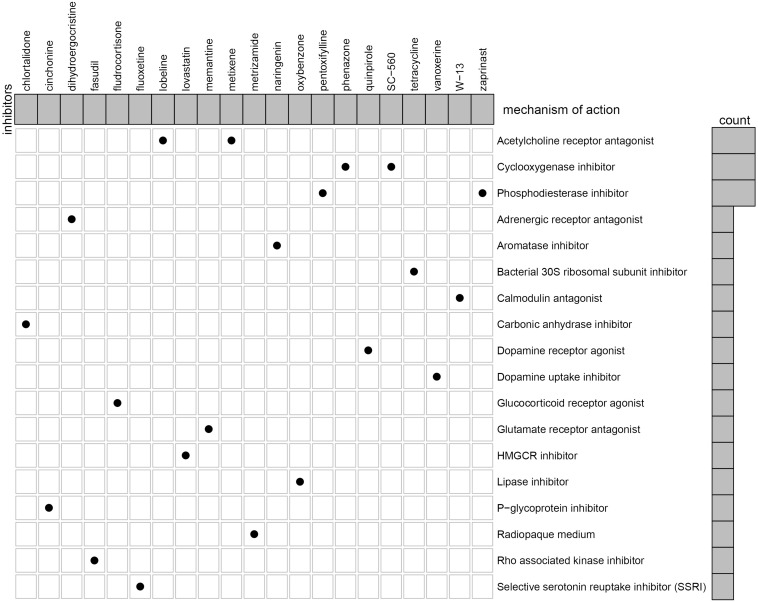
Identification of the small molecular compounds and its mechanism of action for the treatment of EM.

We further identified potential therapeutic drugs for EM treatment by screening the identified DEGs against the CMAP database. Results revealed a total of 47 potential small molecular compounds. Analysis of the mechanism of action of each drug revealed that 21 drugs, namely, W-13, phenazone, metixene, cinchonine, tetracycline, dihydroergocristine, quinpirole, lovastatin, memantine, metrizamide, naringenin, pentoxifylline, lobeline, zaprinast, chlortalidone, SC-560, oxybenzone, fasudil, fluoxetine, fludrocortisone, and vanoxerine, have the capacity for treating EM (Figure 10).

## Discussion

In the present study, we identified DEGs across four datasets obtained from EM and normal tissues. These DEGs included 233 upregulated and 190 downregulated genes, respectively. GO and KEGG analyses revealed that these DEGs were involved in regulating immune-related signaling pathways, such as T-cell activation and regulation, as well as cell chemotaxis. Results from LASSO analysis revealed three key genes, namely, APLNR, CCL21, and FCGR2A. Among them, APLNR was firstly identified as a class A G protein-coupled receptor in 1993, which contained 380 amino acids. Ho et al. (2017) demonstrated that knocking out APLNR in pregnant mice resulted in PE-like symptoms due to defective placental angiogenesis, and this was accompanied by poor placental angiogenesis, elevated apoptosis, and decreased proliferation. Results from analysis of developmental model organisms have suggested that the Apelin/APJ pathway plays an important role in embryo angiogenesis (Kidoya et al., 2008). For example, dysregulated APLNR was associated with growth and migration, as well as cell cycle progression, in ovarian clear cell carcinoma (OCCC) cell lines (Xu and Shen, 2018). However, nothing is known regarding the role of APELA in EM.

C–C motif chemokine ligand 21, a homeostatic lymphoid chemokine that is widely expressed in fibroblasts, smooth muscles, as well as intravascular, T, and dendritic cells, plays an essential role in mediating activated DCs and T cells to lymph system niches and triggering subsequent immune response to foreign antigens (Lin et al., 2014). A previous study showed that poor differentiation was associated with larger tumor size in patients with CCL21-positive gallbladder cancer. Moreover, positive CBS and CCL21 expression are closely associated with clinical severity and poor prognosis in GBC, suggesting that it can be a marker for diagnosis of AC and SC/ASC type in GBC (Li et al., 2020). A CC chemokine receptor 7 (CCR7) and CCL21 are abundant in oral squamous cell carcinoma (OSCC) tissues, where they regulate EMT process and promote OSCC desiccation through activation of the JAK2/STAT3 signaling pathway. CCR7 expression has been associated with poor prognosis of OSCC, while CCL21/CCR7 may be an effective target for prevention and treatment of the condition (Chen et al., 2020). Previous evidence has shown that CCL21 enhances GC progression *via* the MALAT1/SRSF1/mTOR axis, thereby providing a novel therapeutic target for the treatment of GC. Moreover, CCL21-mediated activation of the MALAT1/SRSF1/mTOR axis underpins the development of gastric carcinoma. In fact, Fu et al. (2021) found that CCL21 is significantly upregulated in the EM relative to normal tissues, which is consistent with our results.

The human FCGR2A gene contains at least 800 single-nucleotide polymorphisms (SNPs), located on chromosome 1q23.3 (Velissari et al., 2015). Under physiological conditions, activated FCGriia is constitutively expressed in platelets, neutrophils, macrophages, and dendritic cells, thereby providing an important link between cellular and humoral immunity through a cross-talk between IgG antibody–antigen complexes (Gillis et al., 2014). In fact, this phenomenon is well documented in a number of autoimmune diseases, including rheumatoid arthritis (Jiménez Morales et al., 2019), autoimmune thyroid disease (Mestiri et al., 2020), and idiopathic nephrotic syndrome in children (Rossi et al., 2017). Moreover, Lee et al. (2016) reported that FCGR2A (rs1801274) polymorphism was associated with better response to anti-TNF-a therapy in patients with spondyloarthropathy, psoriasis, and CD. Previous studies have also demonstrated that FCGR2A is the susceptibility gene for Kawasaki disease (KD), while levels in methylation of its CpG site promoter could act as a crucial biomarker for optimizing intravenous immunoglobulin (IVIG) therapy (Kuo et al., 2015). To date, however, the relationship between FCGR2A and EM remains unknown.

In addition, we also evaluated the landscape of 22 immune cells’ infiltration level of EM and normal samples. We found that the T follicular helper cells, gamma delta T cells, resting NK cells, activated NK cells, and M2 macrophages showed a significant divergence between normal and EM tissues. Previous studies have shown that healthy women and EM patients exhibit the largest population of immune cells in their peritoneal fluid. For example, a transcriptome analysis revealed 10,280 and 7,250 single-cell transcripts in the peritoneal fluid of EM and control samples, respectively (Zou et al., 2021). The cells in the peritoneal fluid play a role in immunity and are responsible for removing reflow menstrual debris as well as tissue defense. On the other hand, macrophages represent the largest immune population in the peritoneal fluid, followed by T and dendritic cells, which is consistent with previous studies (Guo et al., 2020). Pillarisetty et al. (2005) reported that other cell groups, including NK, mast, plasma, and epithelial cells, are an intermediate cell type, and named them NKDCs.

The macrophages, primary defense cells in the peritoneal cavity, mainly function to remove cell debris. Notably, EM cells in the peritoneal cavity are not targeted for clearance by phagocytes and NK cells; hence, they are able to escape and survive to invade the peritoneal cavity, *via* a mechanism called “immune escape” (Vetvicka et al., 2016). Vallvé-Juanico et al. (2019) demonstrated that the number and activation of macrophages are high in EM samples, and this was attributed to phagocytosis that clears red blood cells, damaged tissue fragments, and cell debris, and also produces soluble mediators, such as cytokines, prostaglandins, complement components, and enzymes, to regulate the peritoneal environment. Secretion of these immune mediators causes macrophages to induce inflammation, tissue repair, and neovascularization, a phenomenon that may contribute to recruitment of fibroblasts and endothelial cells (Yang et al., 2017). Previous studies have shown that macrophage-derived cytokines stimulate activation of other immune cells, such as T and B lymphocytes. Notably, numerous studies have demonstrated that macrophages are dysfunctional in EM despite an increase in their activation (Zou et al., 2021). Macrophages are functionally differentiated into M1 and M2 cell lines, in the presence of infectious microorganisms or host mediators (Thiruchelvam et al., 2012). Specifically, M1 macrophages are induced by expression of nitric oxide synthase (iNOS) and TNF to produce a large amount of nitric oxide, which is necessary for eliminating bacteria, viruses, and fungi infections. On the other hand, M2 macrophages, also called activated macrophages, respond to parasitic infection and tissue remodeling, and play an important role in angiogenesis and tumor progression. To date, the actual phenotypes of macrophages in the peritoneal fluid in EM patients remains controversial. Recent studies have shown that EM pathogenesis is a complex and heterogeneous phenomenon (Guo et al., 2020). In our analysis, we analyzed the level of 22 immune cells’ infiltration in EM using the CIBERSORTx algorithm and found that M2 macrophages were significantly elevated in EM, relative to normal tissues, indicating that the M2 macrophages might be involved in the pathogenesis of EM.

The NK cells are an important part of the innate immune system, where they play a crucial role in anti-tumor immunity. Some research evidence have shown that NK cytotoxicity increases in the peritoneal fluid of EM patients, with disease severity (Zou et al., 2021). Notably, these changes may interfere with the monitoring, recognition, and destruction of ectopic endometrium cells, thereby causing EM (Gogacz et al., 2017). In our result, we found that the level of resting NK cell and activated NK cell were significantly decreased from the healthy sample to the EM, which may increase the NK cytotoxicity level of EM.

Currently, a lack of non-invasive, clear, and consistent biomarkers for identification of EM have significantly affected accurate diagnosis of the disease. Notably, all pharmacological treatments for EM are inhibitory rather than radical, with non-steroidal anti-inflammatory drugs (NSAIDs) used as the drug of choice for management of EM-related pain (Wattier, 2018). To date, selective progesterone receptor modulators (SPRMs) (Song et al., 2018), oral contraceptives (Grandi et al., 2019), mifepristone (RU486) (Song et al., 2018), Levonorgestrel system (LNGN-ius) (Song et al., 2018), and aromatase inhibitors (Suardika and Pemayun, 2018) have been employed in treating EM. However, their role in restoring fertility and efficacies remains unsatisfactory due to numerous side effects. Here, we investigated the potential drugs through the CMAP dataset to the treatment of EM. We analyzed the mechanism of action of each drug and revealed 21 drugs, namely, W-13, phenazone, metixene, cinchonine, tetracycline, dihydroergocristine, quinpirole, lovastatin, memantine, metrizamide, naringenin, pentoxifylline, lobeline, zaprinast, chlortalidone, SC-560, oxybenzone, fasudil, fluoxetine, fludrocortisone, and vanoxerine, with the potential for treating EM.

This study had a few limitations. Firstly, although we identified and validated potential DEGs, we did not experimentally evaluate their efficacy. There is a need for multi-center prospective studies to evaluate their application. Secondly, we did not elucidate the underlying molecular mechanisms using *in vivo* and *in vitro* experiments. Finally, we did not obtain sufficient clinical information for the subjects herein; therefore, the clinical value of our findings needs further verification.

In conclusion, we identified three key genes between EM and healthy tissue, which may serve as potential biomarkers to the EM. We also evaluated the relationship between key genes and immune cell to explore the molecular mechanism of EM. Finally, we discovered molecular compounds that may contribute to the treatment of EM.

## Data Availability Statement

The original contributions presented in the study are included in the article/Supplementary Material, further inquiries can be directed to the corresponding author/s.

## Author Contributions

YP conceived and designed the experiments and revised and wrote the manuscript. CP carried out the investigation and collected materials. ZF analyzed the data. GC provided administrative, technical, and material support and provided a critical review of the intellectual content of the manuscript. All authors contributed to the article and approved the submitted version.

## Conflict of Interest

The authors declare that the research was conducted in the absence of any commercial or financial relationships that could be construed as a potential conflict of interest.

## Publisher’s Note

All claims expressed in this article are solely those of the authors and do not necessarily represent those of their affiliated organizations, or those of the publisher, the editors and the reviewers. Any product that may be evaluated in this article, or claim that may be made by its manufacturer, is not guaranteed or endorsed by the publisher.

## Footnotes

1
http://r-project.org/

2
https://www.ncbi.nlm.nih.gov/geo/

3
https://bioconductor.org/packages/release/bioc/html/sva.html

4
https://string-db.org/

5
https://cran.r-project.org/web/packages/pROC/index.html

6
https://cibersortx.stanford.edu/

7
https://portals.broadinstitute.org/cmap/

## Supplementary Material

Supplementary Figure 1 ROC analysis was performed to demonstrate the diagnostic power of the three genes in the two external validation datasets.
