# Corrigendum: Bioinformatics Analysis Identififies Molecular Markers Regulating Development and Progression of Endometriosis and Potential Therapeutic Drugs

**DOI:** 10.3389/fgene.2021.793656

**Published:** 2022-01-05

**Authors:** Ying Peng, Cheng Peng, Zheng Fang, Gang Chen

**Affiliations:** Department of Obstetrics and Gynecology, The First Affiliated Hospital of USTC, Division of Life Sciences and Medicine, University of Science and Technology of China, Hefei, China

**Keywords:** endometriosis, bioinformatics analysis, differentially expressed genes, immune mechanism, molecular markers, potential drugs

In the published article, there was an error in the affiliation. Instead of “Division of Life Sciences and Medicine, Department of Obstetrics and Gynecology, The First Affiliated Hospital of USTC, University of Science and Technology of China, Hefei, China” it should be “Department of Obstetrics and Gynecology, The First Affiliated Hospital of USTC, Division of Life Sciences and Medicine, University of Science and Technology of China, Hefei, China”.

The authors apologize for this error and state that this does not change the scientific conclusions of the article in any way. The original article has been updated.

