# Supplementary figures and images for: Bioinformatics Analysis Identifies Molecular Markers Regulating Development and Progression of Endometriosis and Potential Therapeutic Drugs
Source: Front Genet. 2021 Aug 4;12:622683. doi: 10.3389/fgene.2021.622683 (PMC8372410; doi:10.3389/fgene.2021.622683)

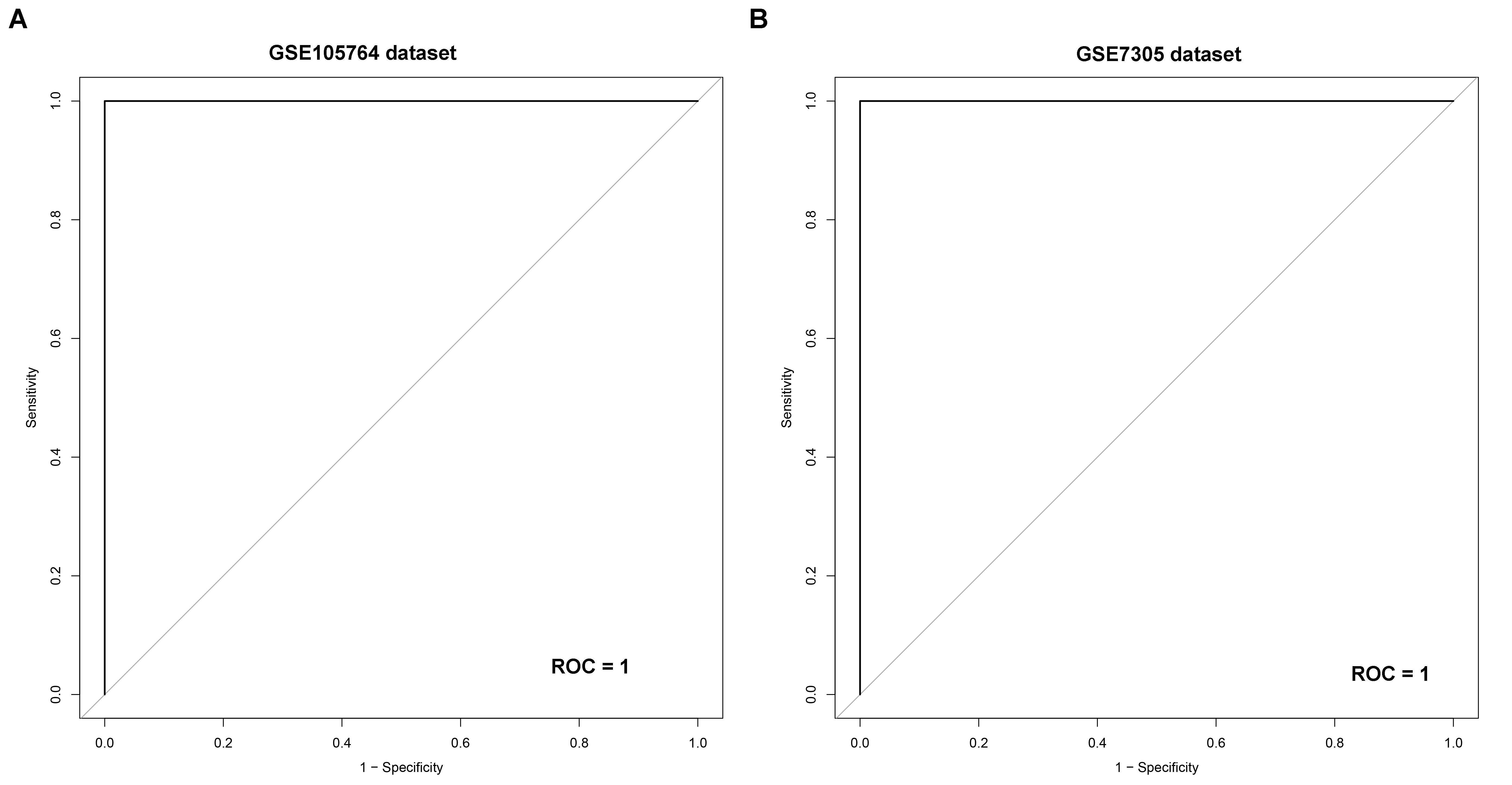

Supplement: Supplementary Figure 1 — ROC analysis was performed to demonstrate the diagnostic power of the three genes in the two external validation datasets. [file Image_1.JPEG]
